# “Upstream Analysis”: An Integrated Promoter-Pathway Analysis Approach to Causal Interpretation of Microarray Data

**DOI:** 10.3390/microarrays4020270

**Published:** 2015-05-21

**Authors:** Jeannette Koschmann, Anirban Bhar, Philip Stegmaier, Alexander E. Kel, Edgar Wingender

**Affiliations:** 1 geneXplain GmbH, D-38302 Wolfenbüttel, Germany; E-Mails: jeannette.koschmann@genexplain.com (J.K.); philip.stegmaier@genexplain.com (P.S.); alexander.kel@genexplain.com (A.K.); 2 Institute of Bioinformatics, University Medical Center Göttingen, D-37077 Göttingen, Germany; E-Mail: anirban.bhar@bioinf.med.uni-goettingen.de

**Keywords:** microarray data, gene expression signatures, upstream analysis, promoter analysis, pathway analysis

## Abstract

A strategy is presented that allows a causal analysis of co-expressed genes, which may be subject to common regulatory influences. A state-of-the-art promoter analysis for potential transcription factor (TF) binding sites in combination with a knowledge-based analysis of the upstream pathway that control the activity of these TFs is shown to lead to hypothetical master regulators. This strategy was implemented as a workflow in a comprehensive bioinformatic software platform. We applied this workflow to gene sets that were identified by a novel triclustering algorithm in naphthalene-induced gene expression signatures of murine liver and lung tissue. As a result, tissue-specific master regulators were identified that are known to be linked with tumorigenic and apoptotic processes. To our knowledge, this is the first time that genes of expression triclusters were used to identify upstream regulators.

## 1. Introduction

Gene activity signatures provide the optimal bar code to characterize the kind and status of a living system (cell, tissue, organ or organism). Thousands of expression experiments have been published and deposited so far in databases such as ArrayExpress [1] or Gene Expression Omnibus (GEO) [2], and derived expression signatures can be found in more specialized databases such as the Expression Atlas [3], the Mouse Expression Database (GXD) [4] or BioGPS [5], to name a few. These signatures can be used as they are, just as a marker for a certain phenomenon of interest, e.g., as biomarker of a specific disease [6]. For a more refined inspection of the biological semantics of the observed expression pattern, differentially expressed genes (DEGs) are identified by comparing gene activity spectra of the cellular system of interest and a control cell. Since the regulation of gene expression, mainly at the transcriptional but also at post-transcriptional level, is involved in nearly any biological process, most standard analyses of transcriptome data usually comprise mapping of DEG sets to Gene Ontology (GO) categories, for instance by GSEA (gene set enrichment analysis) [7]. Regulatory or metabolic pathways that are enhanced by the DEGs can be identified by mapping them, for instance, onto the KEGG pathway database [8]. 

These conventional approaches, which we call “downstream analysis,” give relevant insights into the effects that the induced genes will result in. Since on the other hand they will provide only a very limited clue to the causes that provoke the observed effects, we introduced a novel strategy, the “upstream analysis” approach enabling a causal interpretation of the observed expression changes [9,10,11]. This comprises a state-of-the-art analysis of the promoter structures of the identified DEGs, infers the involved transcription factors (TFs), and identifies the signaling pathways that activate these TFs. In a final step, convergence points of these pathways are identified as potential master regulators or key nodes. Specifically to document pathways regulating the activities of transcription factors and thus enabling this kind of upstream analysis has been the *raison d’être* of the TRANSPATH database, one of the first signaling pathway databases available, which therefore was the optimal source for the analyses reported here [12,13].

Toxic substances exert their effects by affecting a number of pathways, by far not all of them well understood yet. For instance, naphthalene, formerly the main agent in mothballs, is known to cause damages to red blood cells upon long-term exposure [14,15]. The risk caused by naphthalene exposure has been under study since 1980 [16]. It has been found that naphthalene may cause confusion, nausea, vomiting, diarrhea and blood in the urine [16]. Long-term inhalation of naphthalene exerts tumorigenic effects in rats and mice, and in particular female mice showed an enhanced risk to develop alveolar and bronchiolar adenomas of the lung [16].

In this paper, the enhanced upstream analysis was validated by applying it on several toxicologically relevant datasets in order to find out whether naphthalene acts in the two mainly affected tissues, liver and lung, by the same or through different pathways. For this, we have developed and jointly applied novel tools, among them an improved version of our triclustering algorithm δ-TRIMAX [17], which allows for overlapping clusters and minimizes the risk of being trapped in local minima, and a new method to identify enriched transcription factor binding sites in a set of promoters as well as a new master regulator score in the network analysis. Our study revealed that our specific approach of “upstream analysis” was able to identify a number of master regulators for the genes whose activities were affected by intoxication with naphthalene. Among them were some that are known to play essential roles in apoptosis or cancer development. As a conclusion, we propose a novel combination of triclustering with integrated promoter/pathway upstream analysis as a promising approach to identify co-regulated genes and their master regulators.

The workflows used here are freely accessible online on the geneXplain platform [18].

## 2. Experimental Section 

### 2.1. Microarray Data, Differential Expression Analysis

Public datasets from Gene Expression Omnibus (NCBI, Bethesda, MD, USA) were selected to investigate naphthalene effects on different organs/tissues. Experiment GSE18858 is about naphthalene exposure of mouse liver [19] and GSE17933 is about naphthalene exposure of mouse lung [20].

Raw data of naphthalene and control slides were normalized and background corrected using RMA (Robust Multi-array Average). The Limma (Linear Models for Microarray Data) method was applied to define fold changes of genes and adjusted *p*-values. The Limma (Linear Models for Microarray Data) method was applied to define fold changes of genes and to identify the significantly expressed genes using a Benjamini-Hochberg adjusted *p*-value cutoff (≤0.05) [21].

### 2.2. Triclustering of Genes in Expression Data

In order to identify the genes with similar expression profiles over a subset of replicates and a subset of doses of chemical compounds, we have applied an improved version of δ-TRIMAX algorithm [17], called EMOA-δ-TRIMAX (Evolutionary Multi-objective Optimization Algorithm for δ-TRIMAX). It uses a novel Mean Squared Residue (MSR) score as a coherence measure of the resultant triclusters and aims at finding overlapping triclusters from 3D gene expression dataset [22]. The aim is to find large and maximal triclusters, having a MSR score below a certain threshold. In gene expression data, the program thus groups genes according to similarity of their expression levels over multiple doses/time points, as well as samples (*i.e.*, biological replicates). Subsequently, we have identified the genes that are expressed at significantly higher or lower levels at the clustered doses relative to the controls for further analysis using Limma as described above (Section 2.1).

### 2.3. Analysis of Enriched Transcription Factor Binding Sites

Transcription factor binding sites in promoters of differentially expressed genes were analyzed using known DNA-binding motifs described in the TRANSFAC^®^ library, release 2014.4 (BIOBASE, Wolfenbüttel, Germany) [23]. The geneXplain platform provides tools to firstly identify a set of important motifs with occurrences that are enriched in the study promoters as compared to a suitable background sequence set, e.g., composed of promoters whose genes were not differentially regulated in the condition of the experiment. In the following, we denote study and background sets briefly as Yes and No sets. The algorithm for transcription factor binding site (TFBS) enrichment analysis has been described in Kel *et al.* [9]. For each library motif, the procedure finds a score threshold that optimizes the Yes/No ratio *R_YN_* as defined in Equation (1) under the constraint of statistical significance.


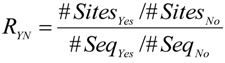
(1)

In Equation (1), *#Sites* and #*Seq* are the sites and sequences counted in Yes and No sequences. A higher Yes/No ratio indicates stronger enrichment of binding sites for a motif in the Yes sequences. One may count all binding sites that occur at a certain threshold and calculate a statistical significance using the one-tailed binomial test

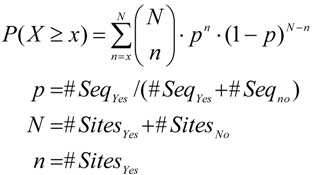
(2)
or one can count only one site for at least one occurrence per sequence and apply the one-tailed Fisher test

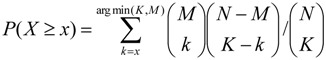
(3)
where *K* denotes the number of sequences with at least one site, *k* are the Yes sequences with a site and *M* = #*Seq_Yes_*. To statistically correct the Yes/No ratio in order to achieve a better ranking of motifs according to their importance, Stegmaier *et al.* [11] described an extension that makes use of the Beta ratio distribution. For improved computational speed, the algorithm incorporated in the geneXplain platform corrects the Yes/No ratio to the lower bound of a chosen confidence interval assuming that the log-Yes/No ratio approximately has a normal distribution [24].

(4) $$\;R_{YN}^{99\%}=\exp\left(\log\left(R_{YN}\right)-\;\alpha_{99\%}∙SE\right)$$

(5) $$SE=\;\sqrt{\frac{1}{\#Sites_{Yes}}+\frac{1}{\#Sites_{No}}+\frac{1}{\#Seq_{Yes}}+\frac{1}{\#Seq_{No}}}$$

**Figure 1:**
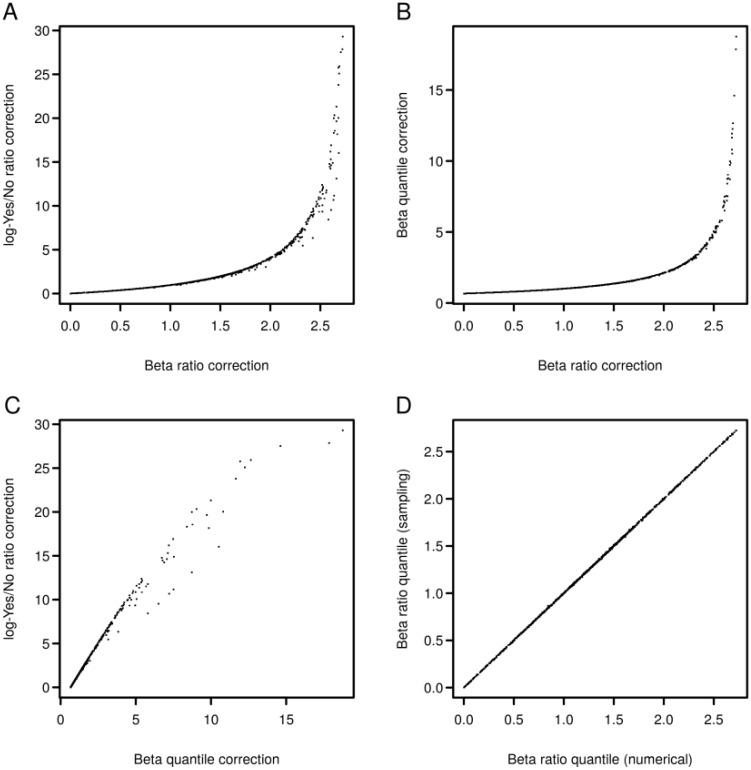
Comparison of different methods for Yes/No ratio correction. (**A**) Beta ratio correction [10] *versus* log-Yes/No ratio correction Equation (4). (**B**) Beta ratio correction *versus* Beta quantile correction Equation (6). (**C**) Beta quantile correction *versus* log-Yes/No ratio correction. (**D**) Comparison of numerical calculation of Beta ratio quantiles to sampling-based quantile estimates.

For the 99%-confidence interval, the geneXplain platform uses an α-value of ~2.576. As an alternative to this approximation and to the Beta ratio-approach [11], one can calculate

(6) $$R_{YN}^{99\%,Beta}=\frac{\#Seq_{No}}{\#Seq_{Yes}+\#Seq_{No}}/Q_{Beta}(.99;\alpha =\#Sites_{No}+1,\;\beta =\#Sites_{Yes}+1)$$

where Q_Beta_ is the quantile function of the Beta distribution. This formula makes use of the Beta distribution for the site proportions whereas the sequence proportion is treated as constant. To our knowledge, these are currently the only described methods that provide a correction for the Yes/No ratio. The speed gain of Equations (4) and (5) over numerical calculation of the quantile of the Beta ratio distribution as described in [11] is substantial. We randomly sampled 1000 parameter sets, each with two values in the interval [1,200] representing binding site counts and two values, 500 and 1000, representing Yes and No sequences. Correction of the log-Yes/No ratio Equation (4), using the Beta distribution quantile Equation (6) or the ratio of Beta distributions [10] for the 1000 parameter sets required, respectively, 0.1 ± 0.008 ms, 10.02 ± 0.14 ms and 19813.5 ± 263.75 ms. Equations (4) and (6) have the additional advantage that their values are not bounded by the relative proportion of Yes sequences. Figure 1A–C compare values returned by the methods for the same parameter sets. The plots show that corrected ratios of all three methods are correlated, where the log-Yes/No ratio correction features some dispersion compared to the methods involving the Beta distribution (Figure 1A,C). This is likely caused by the regularization with a uniform Beta(1,1) distribution. Figure 1D compares Beta ratio quantile values computed numerically for the random parameter sets to sample quantiles obtained by drawing 10,000 samples from corresponding Beta distributions and demonstrates the accuracy of the numerical implementation. 

**Figure 2:**
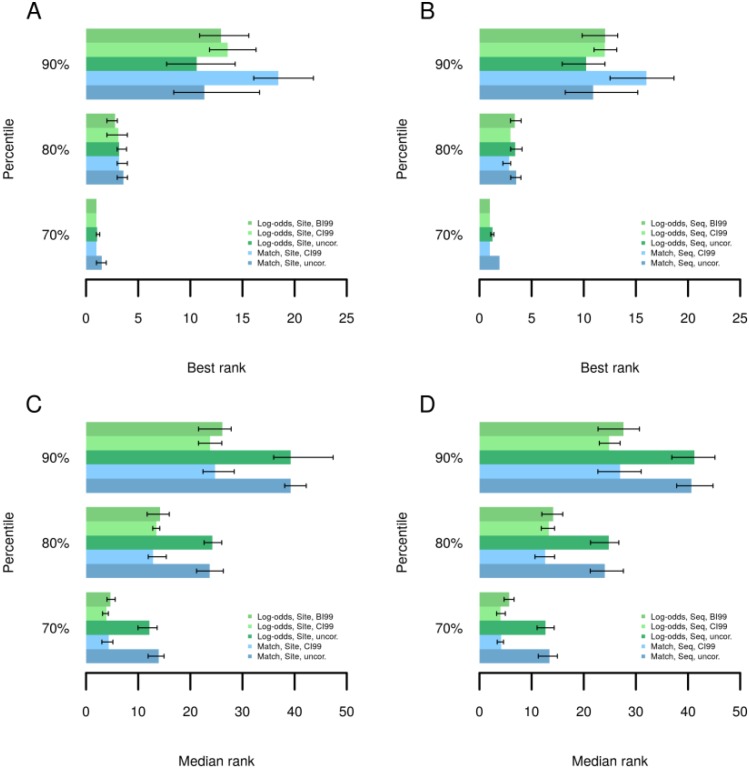
Best and median ranks of known motifs at 70th, 80th and 90th percentiles. ChIP-seq datasets were ordered by observed best or median ranks of motifs known for respective target TFs. Log-odds: Binding sites scored using Log-odds scores; Match: Binding sites scored using MATCH [26] scores; CI99: Correction with confidence interval of 99% as in Equation (4); BI99: Correction based on the Beta quantile function as in Equation (6); Site: Enrichment accounted for all binding sites; Seq: Enrichment accounted for sequences with at least on site. (**A**) Best ranks for site enrichment (**B**) Best ranks for sequence enrichment (**C**) Median ranks for site enrichment (**D**) Median ranks for sequence enrichment.

In the following, we briefly describe how we validated the performance of this method on the basis of experimentally determined transcription factor binding sites. In over 200 ChIP-seq datasets from the Encode project [25] we have determined the ranks of TRANSFAC^®^ motifs corresponding to respective precipitated transcription factors using different methods to calculate Yes/No ratios as well as binding site scores. A method ought to assign a high rank for the true motifs among all motifs of a library. Figure 2 shows that Yes/No ratio correction led to improved or comparable ranking of the best performing motif of a factor in at least 80% of the datasets (Figure 2A,B), where corrections based on Equations (5) and (6) gave similar results. When no method was able to rank the best motif among the first 10 matrices (Figure 2A,B, 90th percentile), then Yes/No ratio correction could decrease the rank of the best motif by about 2–3 positions for Log-odds scores or more strongly for MATCH scores [26]. The low best ranks at the 90th percentile suggest that in these experiments, binding sites of TFs other than the target factor dominated the bound regions and the target TF may have been associated mainly or in some cases by protein-protein interactions only. Comparing the median ranks of motifs for those TFs which are presented by several motifs in the TRANSFAC^®^ database (Figure 2C,D) the corrected Yes/No ratios clearly outperformed the uncorrected ratios in at least 90% of the datasets. The median rank comparison gives an insight into how a method may perform for patterns that do not optimally describe the target TF’s specificity. It can happen that a database comprises only the motif for a related TF or for a more general family or subfamily to which the factor belongs, which may, however, display some differences to the binding properties of the factor of interest. Hence, the Yes/No ratio correction is provided for an improved ranking of motifs for the vast majority of datasets both with regard to the best ranking motif as well as with regard to the entire set of motifs known for some TF. 

In the geneXplain platform, binding site enrichment analysis was carried out as part of a dedicated workflow. The background consisted of 300 house-keeping genes. Promoters were extracted by the workflow with a length of 1100 bp (−1000 to 100).

We considered motifs with corrected Yes/No ratio > 1 for further analysis. The workflow further performs a prediction of binding sites in the promoters of target genes with the filtered matrices at best enrichment cut-offs, maps the matrices to potential transcription factors, and generates visualizations of all results.

### 2.4. Finding Master Regulators in Networks

A second workflow was designed to find master regulatory molecules in signal transduction pathways upstream of identified transcription factors. The workflow firstly maps transcription factors to the TRANSPATH^®^ network (BIOBASE) [13] where they are subjected to a master regulator search with a maximum radius of 10 steps upstream of the factor nodes. A new score is assigned to each potential master regulator that reflects its specificity for the downstream effector TFs Equation (7).


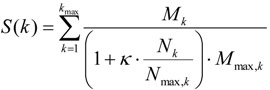
(7)

In Equation (7), *k* is the radius of pathway steps that effector nodes can be separated from the master regulator, *M_k_* is the number of input molecules reached by the regulator within *k* steps, and *N_k_* is the total number of molecules reached from the master regulator within *k* steps. The quantities *M_max,k_* and *N_max,k_* are the highest values among all possible master regulator nodes and normalize the score to the (0,1)-interval. The higher this score, the more specific this master regulator is for the set of input molecules. The parameter *κ* is a user-defined penalty, the default of which is set to 0.1.

To make master regulator scores comparable, we compute a Z-score using 1000 randomly sampled molecule sets of the same size as the input set. These are subjected to the search keeping all other conditions as for the original input. By default, the workflow filters master regulator molecules with Z-score of >1.0 and a score of >0.2. Additional steps are performed by the workflow, such as mapping TRANSPATH^®^ entities to both Ensembl Gene IDs and to UniProt protein IDs. The table with Ensembl Gene IDs is further annotated with additional information, gene description and gene symbols. Finally, the table with master regulatory molecules is sorted by the sum of the ranking of both scores, and networks for each master regulator can be visualized as diagrams in the hierarchical layout.

## 3. Results and Discussion

### 3.1. Integrated Promoter-Pathway Upstream Analysis: Proof of Principle

Our strategy of a causal upstream analysis comprises a systematic and comprehensive promoter analysis of the differentially regulated genes, followed by an analysis of the pathway leading to the regulation of the transcription factors (TFs) involved. Applying this concept in previous studies has successfully revealed EGF and IGF2 as regulators during liver tumor development [11]. More recently and using the workflow components described here, we have identified osteopontin as a key node in the late stage of silicose, when the clinical phenotype becomes manifest [27].

**Figure 3:**
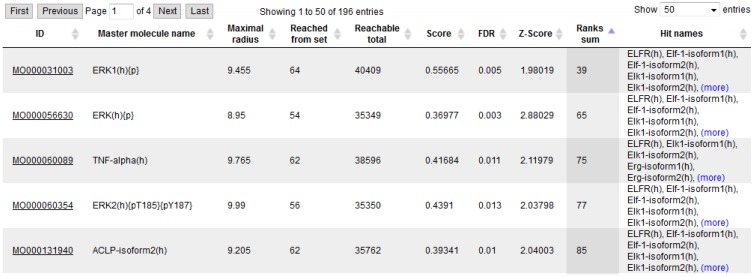
Tabulated top-ranking regulators obtained from an upstream analysis of a TNFα-induced gene set, showing the inducing agent at rank 3.

We also revisited the dataset of TNFα-induced genes in human endothelial cells [28] that we had analyzed in an earlier study showing that the pathways reengineered upstream of these genes and their potential transcriptional regulators pointed to the known TNFα pathway [9]. With the workflow presented here, we could now demonstrate that the inducing agent ranks close to the top of the list of potential master regulators, right under the protein kinase ERK1, a known signal transducer in the TNFα pathway, and its posttranslational modifications and complexes (Figure 3).

### 3.2. Triclustering Identifies Gene Clusters in Three-Dimensional Datasets 

EMOA-δ-TRIMAX identified three gene clusters in the mouse lung profile of Thomas *et al.*, 2009 [20] and 14 gene clusters in the mouse liver profiles of Thomas *et al.*, 2011 [19]. The complete set of clusters is provided in Supplementary Table ST1. For defining the clusters, we first neglected the sign of the gene activity changes (whether up- or down-regulated) and clustered genes with a similar shape of their absolute dose response curves. The rationale behind this is that nearly all regulators (TFs), when put in the appropriate context, can act as transcriptional activators or repressors, either directly or indirectly. It is therefore conceivable that the same regulatory mechanisms are responsible for stimulating the expression of one gene set, but for the repression of another set. Just as a secondary measure, we subdivided the clusters into gene sets that are either up- or down-regulated.

**Figure 4:**
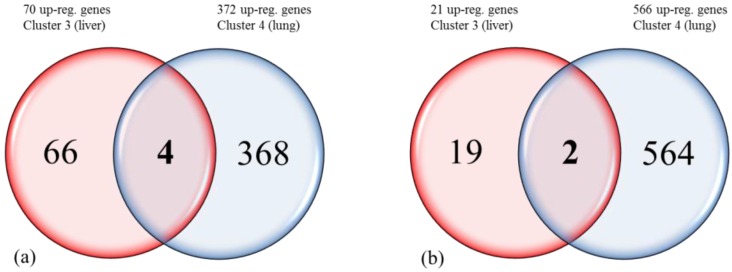
Summary of regulated genes from cluster 3 (red, liver) and cluster 4 (blue, lung) and cluster overlaps. (**a**) Up-regulated genes. (**b**) Down-regulated genes.

We selected cluster 3 from the liver data and cluster 4 from the lung data (gene lists in Supplementary Table ST1) for further analysis, because only these two gene clusters showed similar expression trends in both tissues at the same doses, which were 20 ppm to 30 ppm. The set of up-regulated genes in liver (cluster 3) and lung (cluster 4) comprised 70 and 372 genes of the down-regulated genes in lung and liver comprised 21 and 566 genes, respectively. However, the up- and down-regulated gene sets overlapped in only 4 (up, Fisher test *p*-value: 0.036) and 2 (down, Fisher test *p*-value: 0.11) genes (Figure 4), suggesting that in spite of the observed overlaps, which may be moderately significant for the up-regulated, but hardly significant for the down-regulated genes, naphthalene induces rather specific responses in the two tissues.

The commonly up-regulated genes of both clusters (see Table 1) comprise one alcohol dehydrogenase (Aldh1a3), two proteins involved in GTP-dependent signal transduction (Trio and Gngt1) and one transport protein (Stx6), which may be involved in the metabolism of the toxic compound and its regulation. One of the two commonly down-regulated genes is Vcam1, which is involved in cell-cell adhesion and inflammatory processes. However, all these six common genes show only modest up- or down-regulation, resp.

**Table 1:** Table of common up- or down-regulated genes. Differential expression was quantified in comparison to control replicates.

Ensembl ID	Gene name	Cluster 3	Cluster 3	Cluster 4	Cluster 4
		(log2) fold_change	adj. *p*_value	(log2) fold_change	adj. *p*_value
ENSMUSG00000015134	Aldh1a3	0.436	0.04163	0.164	0.02642
ENSMUSG00000022263	Trio	0.287	0.03742	0.158	0.03514
ENSMUSG00000026470	Stx6	0.444	0.03099	0.137	0.01988
ENSMUSG00000029663	Gngt1	0.582	0.03451	0.178	0.00599
ENSMUSG00000024360	Etf1	−0.732	0.02545	−0.393	0.00802
ENSMUSG00000027962	Vcam1	−0.596	0.03890	−0.151	0.03274

### 3.3. Promoter Analysis

**Figure 5:**
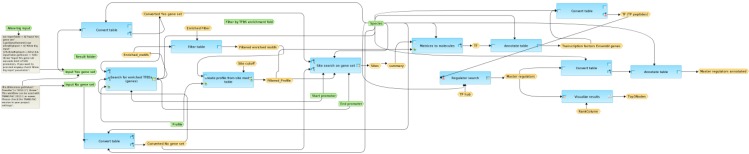
Schematic overview of the workflow “Enriched upstream analysis (TRANSFAC^®^ and TRANSPATH^®^)” with input parameters (green), incorporated and linked (arrows) methods (blue), input/output data (yellow) and additive Javascripts (grey text). See Supplementary Figure SF1 for high-resolution version.

The result of the promoter analysis (see Section 2.2), for which a complex workflow has been composed (Figure 5), comprises enriched TF-binding motifs for each cluster of up- and down-regulated genes. Table 2 lists the transcription factors that were mapped to the identified enriched motifs. Down-regulated genes of cluster 3 (liver) gave 15 potential TFs, and up-regulated genes from the same cluster revealed 17 identified potential TFs. Running the same workflow in parallel for the up- and down-regulated genes of cluster 4 (lung) resulted in the identification of 55 (down) and 68 (up) potential transcription factors. It may be interesting to note that while there is a considerable overlap among the potential regulators of up- and down-regulated genes in the lung (24), up- and down-regulated genes in the liver have no single TF in common; however, these TF lists are also considerably shorter. The up-regulated liver and lung genes share 4 TFs (Egr1, Egr2, Nr2f2/COUP-TF2, Zscan4f); one of them (Egr1) is a known immediate-early response gene, activated by extracellular signals and mediating mitogenic responses [29].

**Table 2:** Table of potential TFs involved in the regulation of the following gene sets: cluster 3 (down-reg. genes in liver), cluster 4 (down-reg. genes in lung), cluster 3 (up-reg. genes in liver) and cluster 4 (up-reg. genes in lung). Underlined are those 4 TFs that are common to the two up-regulated gene sets (liver and lung), two of which appearing in the down-regulated lung set as well.

TFs	TFs		TFs	TFs		
cluster 3	cluster 4		cluster 3	cluster 4		
(liver) down	(lung) down		(liver) up	(lung) up		
Cdx1	Alx1	Lhx1	Ebf1	Alx1	Irf1	Pou2f1
Cdx2	Alx4	Lhx3	Egr1	Arid5a	Irf2	Pou5f1
Hoxc10	Arid3a	Lhx5	Egr2	Ascl1	Irf3	Prdm1
Mafb	Arid5a	Lmx1b	Egr3	Cbfb	Irf4	Prrx1
Mef2a	Bcl6	Nanog	Epas1	Egr1	Irf5	Rara
Pou2f1	Cnot3	Nr2e1	Hivep2	Egr2	Irf6	Rfx2
Pou3f1	Egr2	Otp	Lef1	Foxc1	Irf7	Runx2
Rfx1	Foxa1	Pbx1	Mecp2	Foxf1	Irf8	Runx3
Rfx2	Foxa2	Pbx2	Mtf1	Foxg1	Klf4	Rxra
Rfx3	Foxa3	Pbx3	Myf6	Foxj2	Lhx1	Shox2
Rfx4	Foxc1	Phox2b	Nr2f2	Foxj3	Lhx3	Smad7
Rfx5	Foxd3	Pknox1	Rreb1	Foxk1	Lhx5	Sox12
Six6	Foxf1	Pou2f1	Tcf12	Foxp3	Lhx8	Sox14
Sox21	Foxf2	Prdm1	Tcf7	Gfi1	Lmx1b	Sox21
Tbp	Foxh1	Shox2	Tfap2a	Gfi1b	Meis1	Sox30
	Foxi1	Sox12	Zfp423	Gtf2i	Meis3	Sry
	Foxj1	Sp5	Zscan4f	Hdx	Msx1	Tbx15
	Foxk1	Srebf1		Hnf1a	Msx3	Vsx1
	Foxp3	Stat5a		Hnf1b	Nr2c2	Zfp184
	Gfi1	Stat5b		Hoxa4	Nr2f2	Zfp426
	Gli1	Tcf3		Hoxa9	Pax6	Zfp445
	Gli2	Uncx		Hoxb4	Phox2b	Zscan4f
	Gtf2i	Vsx1		Hoxc4	Pknox2	
	Hoxb4	Zfp30				
	Hoxc4	Zfp784				
	Hoxd8	Zic1				
	Irf1	Zscan4f				
	Irf5					

### 3.4. Find Master Regulators in Networks

**Figure 6:**
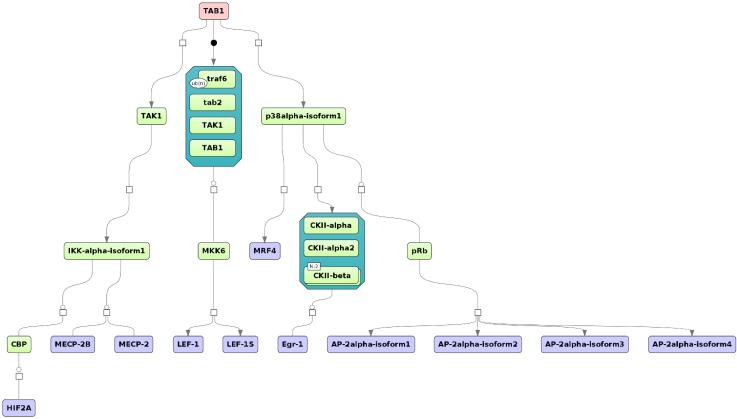
Master regulator TAB1 was identified for the cluster of up-regulated genes in the liver. The master regulator is shown at the top-most position of the schematic overview (pink rectangle), connecting molecules up to 10 steps upstream (green rectangles) starting from the identified transcription factors (blue rectangles). Known complexes are highlighted by the dark-green hexagonal frames. The diagram is a result of the workflow shown in Figure 5. See Supplementary Figure SF2 for high-resolution version.

When we followed the upstream activation pathways of the TFs potentially involved in the (co-)regulation of the liver cluster 3 genes, we found TAB1 as one potential master regulator of the up-regulated genes (Figure 6). Mapping expression values from the whole liver experiment showed no highly up- or down-regulated genes for the involved proteins of the identified pathway.

TAB1 is a protein that binds to and regulates the activity of the mitogen-activated protein kinase MAP3K7, also known as TGF-β-activated kinase 1 (TAK1). This kinase mediates TGF-β and TNF-α signals and, via some phosphorylation events, activates the NF-κB pathway and the MAPK pathways, the latter targeting transcription factor (TF) AP-1 and related TFs. This way, TAK has been shown to play a dual role as both a tumor-promoting and suppressing agent, depending on the cellular context [30,31,32]. In liver, the role of TAK1 as tumor suppressor has been demonstrated [32]. Based on these findings, TAK1 has been discussed as a potential target for cancer treatment [33,34].

**Figure 7:**
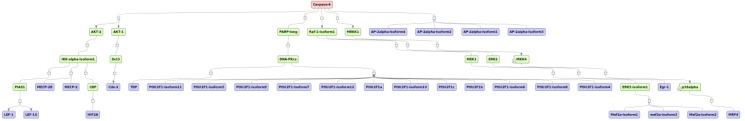
The potential master regulator caspase 6 (pink rectangle) was identified for the cluster of both up-and down- regulated genes in naphthalene-treated mouse liver. The master regulator is shown at the top-most position of the schematic overview (pink rectangle), connecting molecules up to 10 steps upstream (green rectangles) starting from the identified transcription factors (blue rectangles). The diagram is a result of the workflow shown in Figure 5. See Supplementary Figure SF3 for high-resolution version.

In addition, caspase 6 was found to be a common master regulator of up- and down-regulated genes in liver (Figure 7). This gene encodes a cysteine-aspartic acid protease (caspase). Caspases are activated by proteolytic processing cascades [35,36]. Their sequential activation is essential for cell apoptosis [37]. However, caspase 6 seems to be an exception in that its activation does not necessarily depend on other caspases and, thus, its role in apoptosis might be a different one compared to the other caspase family members, subject to further proofs [38].

**Figure 8:**
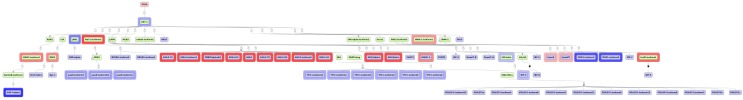
Identified master regulator PTK6 is shown at the top-most position of the schematic overview (pink rectangle), connecting molecules up to 10 steps upstream (green rectangles) starting from the identified transcription factors (blue rectangles). Strong red border lines indicate up-regulated genes and blue border lines down-regulated genes. The diagram is a result of the workflow shown in Figure 5, mapped with expression values. See Supplementary Figure SF4 for high-resolution version.

The upstream strategy applied to the up-regulated genes of lung cluster 4 revealed PTK6 (protein tyrosine kinase 6) as one of the top-most six upstream regulators (Figure 8). Expression mapping showed that many of the identified potential TFs are either up- or down-regulated (blue and red border lines in Figure 7). Down-regulated expression of protein tyrosine kinase 6 (PTK6) is correlated with poor survival in esophageal squamous cell carcinoma [39]. A previous study showed over-expression of PTK6 in non-small-cell lung cancer (NSCLC) and evaluated its pathological and prognostic significance [40]. The results confirmed that NSCLC patients with overexpressed PTK6 had a poor survival prognosis, rendering PTK6 inhibitors candidate drugs for treating this kind of cancer [40].

**Figure 9:**
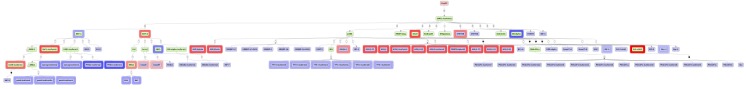
The potential master regulator Usp22 is shown at the top-most position of the schematic overview (pink rectangle), connecting molecules up to 10 steps upstream (green rectangles) starting from the identified transcription factors (blue rectangles). Strong red border lines indicate up-regulated genes and blue border lines represent down-regulated genes. The diagram is a result of the workflow shown in Figure 5, mapped with expression values. See Supplementary Figure SF5 for high-resolution version.

Usp22 was found to be a common master regulator for up- and down-regulated genes in mouse lung. Usp22 encodes ubiquitin carboxyl-terminal hydrolase 22 (Figure 9). As a component of the histone acetylation (HAT) complex SAGA, Usp22 removes the ubiquitin residues from histones H2A and H2B, which leads to a transcriptional (co-)activation [41,42,43]. Human USP22 is known to play a role in different types of cancer [44,45,46]. In particular, it has been demonstrated that overexpression of USP22 is associated with non-small-cell lung cancer (NSCLC) and causes a poor survival prediction [44].

Altogether, we noticed that the suggested master regulators for both tissues are involved in promoting tumor progression and/or apoptosis. Those found in the liver seem to be of a more general function, whereas those identified from the lung dataset have the potential to specifically trigger the development of lung tumors (NSCLC). Thus far, we have not been able to directly compare the results of our analysis with what other tools aiming at upstream analyses would result in, such as IPA [47]. It is our aim to model a mechanistically plausible upstream pathway, for which the most crucial first step is the identification of all relevant TF-target gene relations. For this, we apply a *de novo* rather than a knowledge-based strategy. Our approach stresses the importance of regulation through TF combinations and secures the required flexibility for the analysis of new cellular systems, e.g., tumors that have not yet been studied and in which the existing TF repertoire has usually been redirected to govern a significantly different genetic program, e.g., as described in [48]. Optimally, each newly studied cellular system would be experimentally characterized for genomic locations of all ~1600 TFs (in case of mammals), as was done exemplarily for one TF (BCL6) in a previous study [49], which is not yet feasible. We therefore feel that our approach represents a good and realistic compromise between reliable knowledge-based pathway reengineering and flexible *de novo* analysis of regulatory genome regions.

## 4. Conclusions 

We have outlined our strategy of “upstream analysis,” which is an integrated promoter and pathway analysis. The largest part of this analysis has been put together as a workflow in the geneXplain platform. Part of its efficiency is due to a novel approach to identify enriched transcription factor binding sites, which improves the ranking of true motifs according to the (corrected) Yes/No ratio, specifically for suboptimal motif patterns as validated on a large number of ChIP-seq datasets. Here we present two formulas to calculate the correction which provide substantial speed improvements over our previous method. We have compared different methods to obtain the best ranking of motifs and found that Yes/No ratio correction improves the ranking of true motifs, where the confidence interval-based correction is simple to compute and performed comparably to a method making use of the Beta distribution. When we applied our strategy to clustered gene sets of liver or lung tissue that were exposed to a toxicant (naphthalene), we were able to identify tissue-specific targets and master regulators. In the case of liver, these master regulators indicate that some general tumor and apoptosis-promoting pathways may be triggered, whereas in the lung tissue, master regulators were found that specifically trigger aggressive lung cancer to develop. These results demonstrate the validity of the presented upstream analysis strategy.

## Supplementary Files

Supplementary File 1
