# Supplementary figures and images for: “Upstream Analysis”: An Integrated Promoter-Pathway Analysis Approach to Causal Interpretation of Microarray Data
Source: Microarrays (Basel). 2015 May 21;4(2):270–86. doi: 10.3390/microarrays4020270 (PMC4996392; doi:10.3390/microarrays4020270)

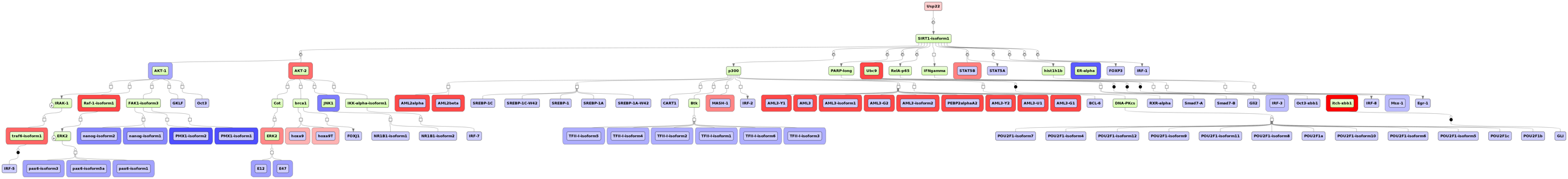

Supplement: Supplementary File 1 [file microarrays-04-00270-s001.zip › SF5.png]

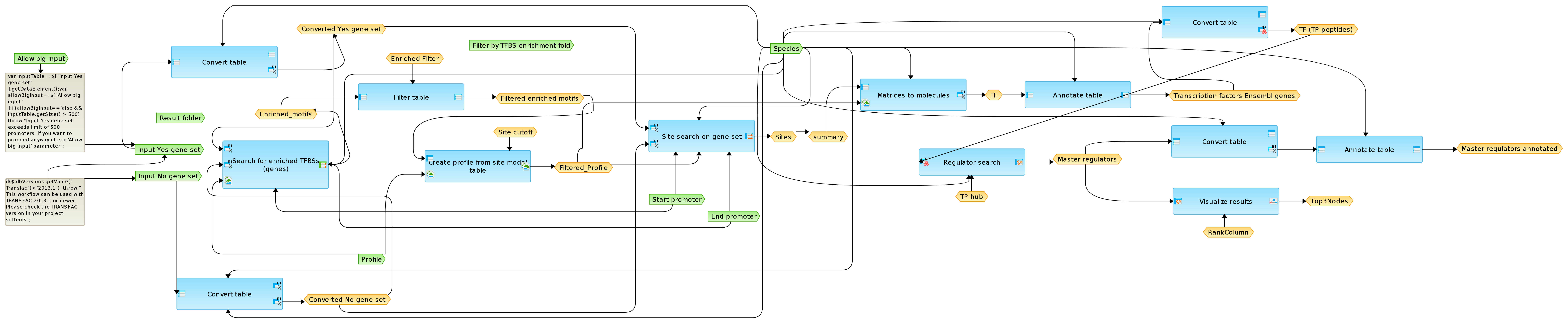

Supplement: Supplementary File 1 [file microarrays-04-00270-s001.zip › SF1.png]

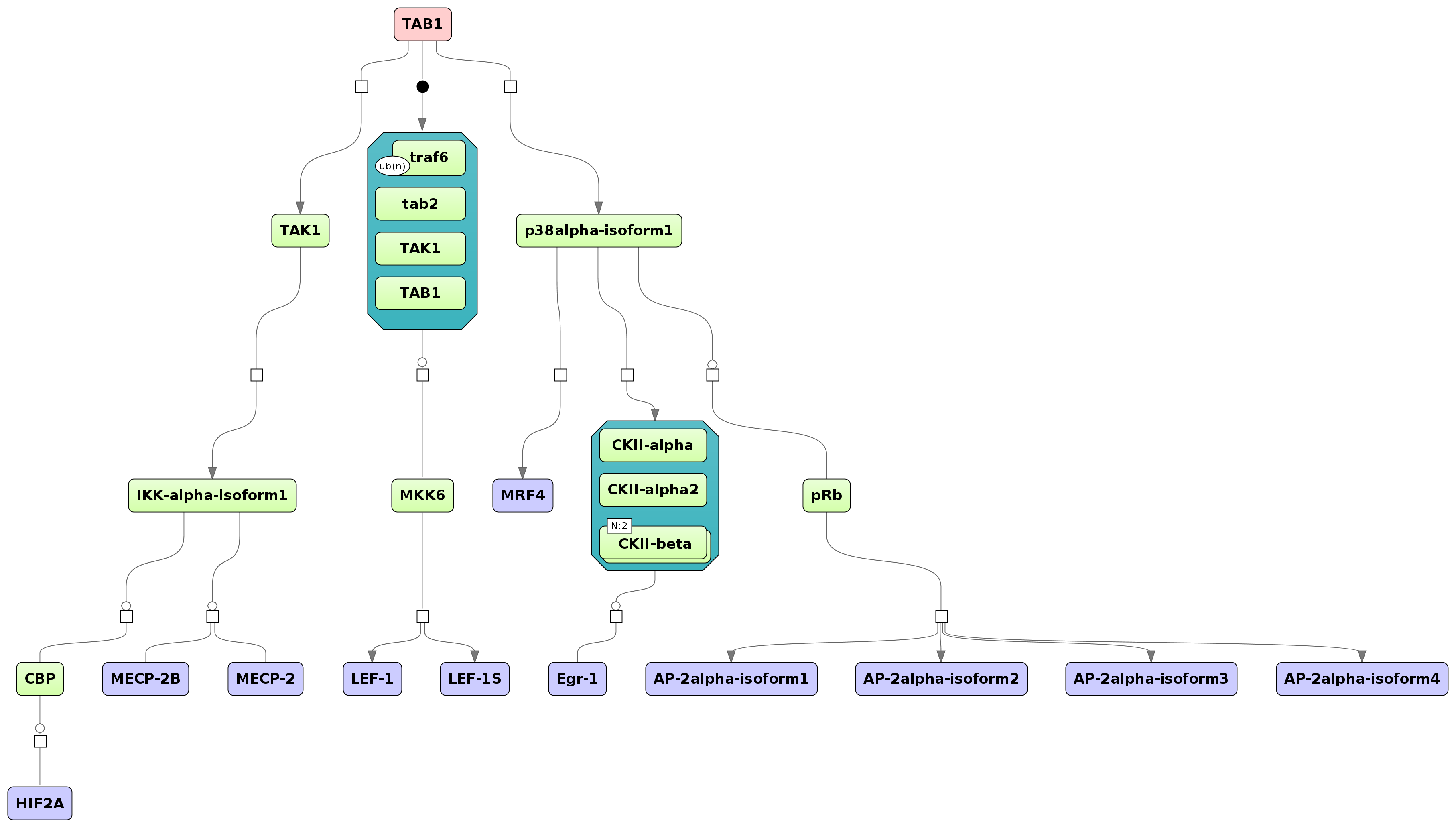

Supplement: Supplementary File 1 [file microarrays-04-00270-s001.zip › SF2.png]

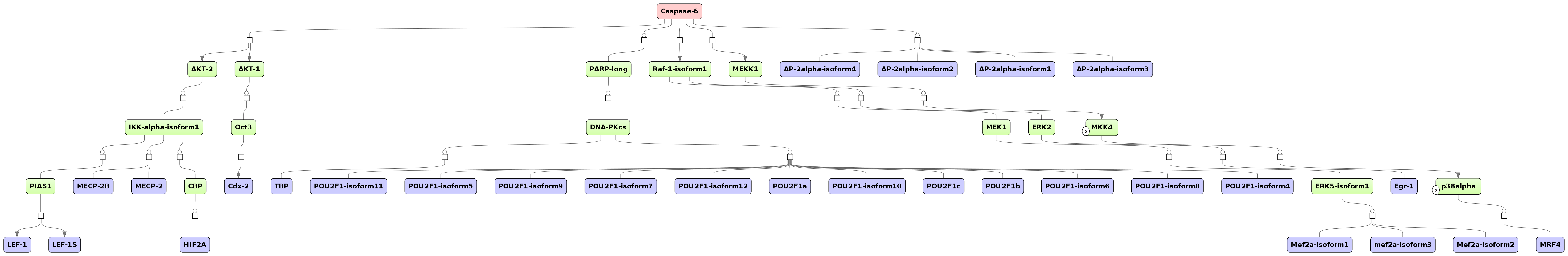

Supplement: Supplementary File 1 [file microarrays-04-00270-s001.zip › SF3.png]

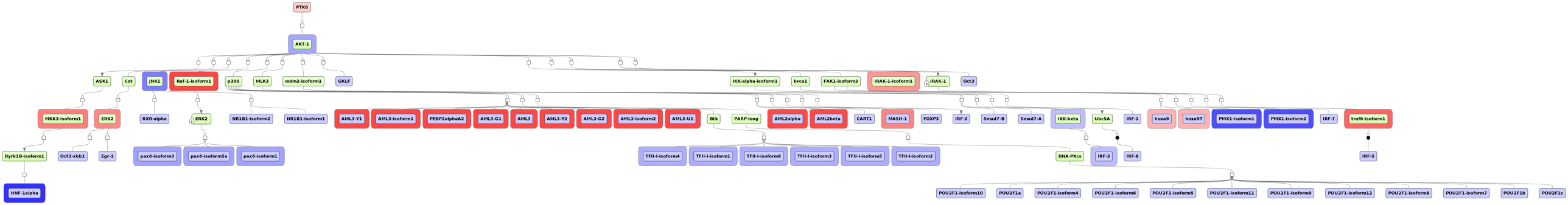

Supplement: Supplementary File 1 [file microarrays-04-00270-s001.zip › SF4.png]
